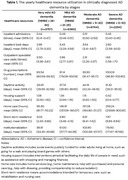# Healthcare resource utilization of Alzheimer's disease – a Swedish quality register‐based cohort study

**DOI:** 10.1002/alz70860_102643

**Published:** 2025-12-23

**Authors:** Xin Xia, Alice Clark, Niels Juul Brogaard, Jens Gundgaard, Pepa Polavieja, Maria Eriksdotter, Henrik Zetterberg, Silke Kern, Tobias Skillbäck, Linus Jönsson

**Affiliations:** ^1^ Karolinska Institutet, Solna, Stockholm, Sweden; ^2^ Novo Nordisk A/S, Søborg, Denmark; ^3^ Karolinska Institutet, Huddinge, Stockholm, Sweden; ^4^ Karolinska University Hospital, Stockholm, Sweden; ^5^ University of Gothenburg, Mölndal, Sweden; ^6^ Sahlgrenska University Hospital, Mölndal, Sweden; ^7^ University College London, London, United Kingdom; ^8^ University of Wisconsin‐Madison, Madison, WI, USA; ^9^ Hong Kong Center for Neurodegenerative Diseases, Hong Kong, Hong Kong, China; ^10^ University of Gothenburg, Gothenburg, Sweden; ^11^ Sahlgrenska University Hospital, Mölndal, Gothenburg, Sweden

## Abstract

**Background:**

The study aimed to provide estimates of healthcare resource utilization (HCRU) of clinically diagnosed AD, which can be used as input parameters for economic modeling of future pharmacological treatments for AD.

**Method:**

We used longitudinal data between 2013 and 2020 from the Swedish Register of Cognitive Disorders (SveDem) to identify 20366 individuals (mean age: 78.4 years) with clinically diagnosed AD dementia. AD dementia stages were defined by MMSE as follows: very mild (MMSE = 26‐30), mild (MMSE = 21‐25), moderate (MMSE = 11‐20), and severe (MMSE = 0‐10). SveDem was linked to the following registers to derive data on HCRU for these individuals: (1) the National Patient Register for information on inpatient admissions, inpatient bed days, and outpatient specialist visits, (2) the Drug Prescription Register for information on drug prescriptions, and (3) the Register of social care for elderly and persons with functional disability for information on social care (daytime activity, housing support, homecare, short‐term residence, and institutionalization). We described the HCRU by stages of AD dementia and HCRU categories.

**Result:**

The number of outpatient specialist visits decreased as AD severity increased, while the number of drug prescriptions increased with advancing stages. Social care, especially day activity, homecare, and institutionalization days, evidently increased in more advanced AD stages compared with earlier stages. The use of inpatient care was similar across different stages. The detailed HCRU by AD stages are shown in Table 1.

**Conclusion:**

In people with AD dementia, drug prescriptions and social care utilization increase with advancing AD severity. In contrast, outpatient specialist care utilization decreases with advancing AD severity. The results of this study can serve to provide better understanding on HCRU and the type of care provided to individuals with AD by AD stages and can also be used as input parameters for future economic evaluations of new treatments for AD.